# Effect on Rheological Properties and 3D Printability of Biphasic Calcium Phosphate Microporous Particles in Hydrocolloid-Based Hydrogels

**DOI:** 10.3390/gels8010028

**Published:** 2022-01-02

**Authors:** Helena Herrada-Manchón, David Rodríguez-González, Manuel Alejandro Fernández, Nathan William Kucko, Florence Barrère-de Groot, Enrique Aguilar

**Affiliations:** 1 Centro de Innovación en Química Avanzada (ORFEO-CINQA), Departamento de Química Orgánica e Inorgánica, Instituto Universitario de Química Organometálica “Enrique Moles”, Universidad de Oviedo, C/Julián Clavería 8, 33006 Oviedo, Spain; davidrgezglez@gmail.com (D.R.-G.); eah@uniovi.es (E.A.); 2 Fundación Idonial, Parque Científico y Tecnológico de Gijón, Avda, Jardín Botánico 1345, 33203 Gijón, Spain; alejandro.fernandez@idonial.com; 3 Kuros Biosciences BV, Professor Bronkhorstlaan 10, Building 48, 3723 MB Bilthoven, The Netherlands; Nathan.Kucko@kurosbio.com (N.W.K.); florence.de.groot@kurosbio.com (F.B.-d.G.)

**Keywords:** 3D printing, biphasic calcium phosphate, hydrogels, rheology, solid dispersions

## Abstract

The production of patient-specific bone substitutes with an exact fit through 3D printing is emerging as an alternative to autologous bone grafting. To the success of tissue regeneration, the material characteristics such as porosity, stiffness, and surface topography have a strong influence on the cell–material interaction and require significant attention. Printing a soft hydrocolloid-based hydrogel reinforced with irregularly-shaped microporous biphasic calcium phosphate (BCP) particles (150–500 µm) is an alternative strategy for the acquisition of a complex network with good mechanical properties that could fulfill the needs of cell proliferation and regeneration. Three well-known hydrocolloids (sodium alginate, xanthan gum, and gelatin) have been combined with BCP particles to generate stable, homogenous, and printable solid dispersions. Through rheological assessment, it was determined that the crosslinking time, printing process parameters (infill density percentage and infill pattern), as well as BCP particle size and concentration all influence the stiffness of the printed matrices. Additionally, the swelling behavior on fresh and dehydrated 3D-printed structures was investigated, where it was observed that the BCP particle characteristics influenced the constructs’ water absorption, particle diffusion out of the matrix and degradability.

## 1. Introduction

The working principle of 3D printing is that objects can be created by adding material in a layer-by-layer manner via automated deposition and computer-aided design/computer-aided manufacturing (CAD/CAM) systems. Since the repair of large-area and complex-shape bone defects remains a significant challenge, the 3D printing of on-demand bone graft biomaterials has been emerging as an alternative to autografts in the last few years [1,2,3]. As a replacement to autografts, synthetic bone graft biomaterials are available in unlimited quantities and have the advantage of being sterile and do not carry a risk of disease transfer. Moreover, they have the added benefit of eliminating the need for invasive harvesting of autologous bone, which can lead to donor-site morbidity [4,5,6]. Furthermore, synthetic bone graft biomaterials provide the ability to control the shape, size, surface morphology and internal porosity of the final 3D-printed product, thereby facilitating the ability to deliver fully personalized solutions for repairing difficult, critical-size bone trauma defects.

Amongst all the 3D-printing technologies developed, extrusion-based printing of gels—commonly known as semi-solid extrusion (SSE)—has been widely used due to its exceptional capability of accommodating a diverse range of printable fluids whose viscosities range from 30 mPa s^−1^ to just under 6 × 10^7^ mPa s^−1^ [7,8]. Although considerable work has been done in developing functional ceramic inks and bioinks, i.e., biological cocktails of ceramic materials, cells, growth factors or drugs, among others, many extrudable combinations are only capable of printing simple structures that are only a few millimeters tall due to limitations in flow characteristics [8,9]. More promising results in precisely replicating centimeter-scale bone segments have been obtained with ready-to-use commercial pastes [3,10,11] or formulations loaded with small (<30 µm) spherical powders, which have better fluidity than irregularly shaped powders and are more convenient to 3D print [9,12,13,14]. However, it has been widely reported that surface architecture, geometry, and microporosity are essential factors for osteoconduction (the ability to form new bone on the surface of biomaterials) and, in some cases, osteoinduction (induction of de novo bone formation through cellular differentiation into bone-forming cells) [4,7,15,16,17,18]. Due to the latter, exploring the printability of irregularly shaped, microporous biphasic calcium phosphate (BCP) granules 150–500 μm in size with a characteristic submicron surface structure is of particular interest. BCP consists of a mixture of hydroxyapatite (HA) and beta-tricalcium phosphate (β-TCP), which are materials that are widely used in the field of bone regeneration due to their excellent biocompatibility and osteostimulatory properties that are attributed to their similar chemical composition to the inorganic phase of bone [13,15,17]. Furthermore, the BCP granules used in this study have a characteristic submicron surface structure that has been shown to be osteoinductive and demonstrated enhanced orthotopic bone formation compared to other conventional calcium phosphate bone substitutes [4].

The use of hydrogels as particle carriers is a straightforward approach for the fabrication of bone graft substitutes. Nevertheless, the combination of this hydrocolloid-based vehicle with BCP particles must result in compositions that exhibit a proper rheology, viscosity, and gelation time, for acquiring steady multi-layered final constructs. For SSE 3D printing, shear-thinning and viscoelastic fluids are highly desirable, as they can be easily extruded through a nozzle and rapidly recover the viscosity and mechanical strength necessary to support the next layer extruded [19,20,21,22]. Due to the physical characteristics of the BCP particles (size, weight, and agglomeration trend), three well-known hydrocolloids (sodium alginate, xanthan gum, and type A gelatin) have been combined, exploiting their different intrinsic properties as thickeners and/or gelling agents, to generate stable, homogenous, and printable solid dispersions which, to the best of our knowledge, have not been previously explored. In parallel, in the case of repairing bone defects, accumulating evidence indicates that the matrix stiffness, viscoelasticity, and topography have potent effects on cell behavior, resulting in better osteointegration and osteoinduction [4,23,24]. Therefore, it is necessary to investigate the relationship between the biomechanical properties of the hydrogels in terms of stiffness (elastic modulus), loading percentage of BCP granules, and crosslinking time. In addition, when dealing with 3D-printed constructs, the stiffness will differ according to the configuration of the printing process parameters, such as the infill density and the infill pattern. Taking all this into account, the current work is directed toward the research of new ceramic inks by assessing their rheological properties, stiffness, and mechanical load resistance, which will enable a future development of customized 3D-printed bone grafts for the repair of critical-size bone defects.

## 2. Results and Discussion

### 2.1. BCP-Hydrogel Inks Composition and Formulation

**Figure 1:**
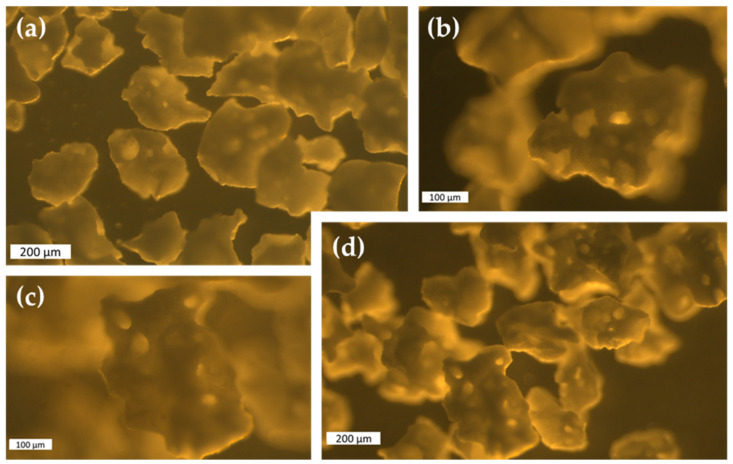
Stereomicroscopic images of 150–250 µm (**a**,**b**) and 150–500 µm (**c**,**d**) BCP granules. Scale bars: 200 µm (**a**,**d**), 100 µm (**b**,**c**).

The observation of the BCP granules revealed irregularly shaped particles with high microporosity and size ranges of 150–250 µm for BCP1 granules and 150–500 µm for BCP2 granules (Figure 1). Printing these particles through SSE required a carrier whose consistency was critical: it had to be fluid enough to be extruded while being thick enough to keep the particles suspended. In that sense, a biocompatible colloidal system was designed for the purpose of loading BCP granules and controlling the rheological properties to ensure good printability. The selection of the materials (sodium alginate, gelatin, and xanthan gum) was according to the formulation needs in terms of thermoreversibility, high affinity for water, and pseudoplastic behavior with high viscosity at rest and stability under shear during processing.

Alginate is a naturally occurring anionic polymer typically obtained from brown seaweed that has been extensively investigated as a hydrogel material for tissue engineering applications due to its favorable properties such as low toxicity, biocompatibility, and easy processability. Through the addition of divalent cations, such as Ca^2+^, alginate undergoes a gelation process where the polymer chains form an “egg-box structure” with the divalent ions, giving immediate rigidity and allowing manipulation of the printed structures. However, the rheological properties of pure alginate result in poor printability and pattern fidelity, which greatly limits its use in 3D printing [25,26,27]. Gelatin, derived from collagen by either acid (type A) or alkaline (type B) hydrolysis, is one of the most extensively used protein hydrocolloids [28,29]. As a counterpoint to its low cost, ease of preparation, and biocompatibility, gelatin undergoes a gel-sol transition at physiological temperature (37 °C) that makes essential the induction of any crosslinking reaction (chemical, light, or pH-dependent) on the printed structures to avoid a rapid destruction in culture conditions and facilitate the handling of the samples [30,31,32]. In this case, gelatin provides the mixture with a thermoreversible behavior, which facilitates the incorporation of the remaining ingredients and the loading process into the syringes by staying as a viscous liquid above 37 °C. Xanthan gum is an anionic heteropolysaccharide produced by the bacteria *Xanthomonas campestris* and traditionally used as a binder, thickener, stabilizer, and bodying agent in confectionary and dairy products, as it has high stability in a wide range of pH, temperature, and ionic strength [29,33,34]. Made up of various monosaccharides, mannose, glucose, and glucuronic acids, macromolecules of xanthan can have the conformation of simple, double, or triple helicoidally flexible chains, which are able to interact to increase the viscosity of the solution and grant a highly shear-thinning behavior of the fluid [33,35]. This pseudoplastic behavior helps in mixing, pumping, filling, and pouring processes. Although xanthan gum is well known for its non-toxicity and excellent biocompatibility [36], its use has not yet been fully exploited for tissue engineering and 3D printing, with only few works being reported in the literature and none of them specifically for bone regeneration [37,38,39]. Xanthan gum provides yield stress and increases viscosity, allowing particles of up to a large size to remain homogeneously suspended within the hydrogel [40]. In general, hydrogels only form if there is enough gelling agent present and the number of junction zones is sufficient to hold the entrained liquid [41]. The combination of these colloids enhances gel formation through protein/polysaccharide interactions—usually dominated by electrostatic forces—molecular entanglement, and ionic bridges. Consequently, the interactions between these biopolymers can be tuned by controlling the pH, the ionic strength, and the protein/polysaccharide ratio [28]. The incorporation of BCP granules in a high proportion in the viscous polymer solution made the mixture become thicker, behaving more similarly to a gum than a gel.

To date, much research has focused on 3D-printing inks loaded with spherical micro or nanoparticles. However, particles that are anisometric introduce several additional effects [42]. The local flow around a spherical particle is different than that of non-spherical particles which, in addition, are orientable, so the contribution to suspension viscosity is also different. Furthermore, particle interactions are strongly influenced by particle shape and, in general, the degree of interaction among non-spherical particles is greater than among spherical particles at the same concentration, as the higher specific surface enhances the probability of interactions [42]. For that purpose, the influence of particle size and proportion on the ink’s behavior was assessed through different rheological tests.

### 2.2. Shear Viscosity Tests and Effect of Particle Loading

**Figure 2:**
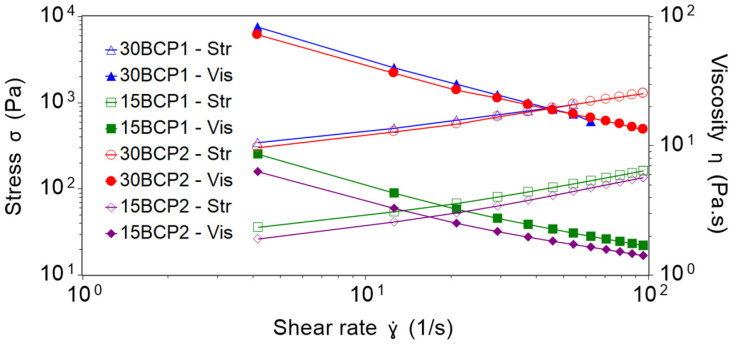
Shear stress (Str) and viscosity (Vis) profiles from 0.01 to 100 s^−1^ within 120 s at 37 °C.

Particle loading is one of the factors that affect the rheology of dispersions and suspensions. Volume fraction (ϕ) measures the volume of particles divided by the total volume of the suspension, and the maximum packing fraction ($\phi_{max})$ corresponds to the maximum volume of particles that could be added to a suspension, which depends on the size and the shape of the particles. The Krieger-Dougherty model (1) describes the relationship between viscosity of the material and volume fraction of solid particles for fluids with high solid content characteristics:

(1) $$\frac{\eta }{\eta_{med}}={\left(1-\frac{\phi }{\phi_{max}}\right)}^{-\left[\eta \right]\phi_{max}},$$

where η is the viscosity of the suspension, and $\eta_{med}$ is the viscosity of the medium [43,44]. Following this model, the viscosity of the suspension is going to be affected by ϕ and $\phi_{max}$. Thus, increasing the volume fraction increases the viscosity, as packing molecules makes flow more difficult. In this manner, the flow curves (Figure 2) reflected higher viscosity values for hydrogels loaded with 30 wt % of BCP (30BCP1 and 30BCP2). Furthermore, all the mixtures showed a decrease in viscosity when increasing shear rates, revealing clear shear-thinning properties of the BCP-hydrogels. This time-independent non-Newtonian fluid behavior is highly convenient for fluids that undergo a high shear condition while passing through a nozzle, as is the case with 3D-printing inks.

Shear-viscosity profiles were similar for inks with the same particle concentration, although the particle size was different. Thus, the profiles of 30BCP1 and 30BCP2 practically overlap and, in turn, 15BCP1 and 15BCP2 have closer values and the same tendency. However, 30BCP1 became shear-thickening at higher shear rates and suffered jamming, spilling the sample out of the rheometer plates and invalidating the measurements. For that reason, 30BCP1 was measured only between 0.01 and 70 s^−1^. This effect did not occur in the case of 30BCP2, verifying the impact of particle size in the rheology of solid dispersions.

The Herschel–Bulkley mathematical model fit the experimental results with R² values higher than 0.999 for all the inks (Table 1). For the same particle concentration, the larger the size of the granules, the higher the yield stress and *K* value, denoting an increase in apparent viscosity. Similarly, the mixtures with a higher quantity of particles had a lower n value, and therefore, a greater pseudoplastic behavior, which was most likely due to the increase in colloidal interactions and particle–particle interactions that are easily shattered with shear.

### 2.3. Thixotropy and Viscosity Recovery Analysis

Thixotropy is a time-dependent shear-thinning property that is used to characterize the structure change reversibility and can be quantitatively measured on a rotational rheometer through a Stepped Flow Method (SFM). With this three-step method, the fluid attains the state of rest in the first step, suffers a structural destruction in step two, and regenerates the structure in step three.

**Figure 3:**
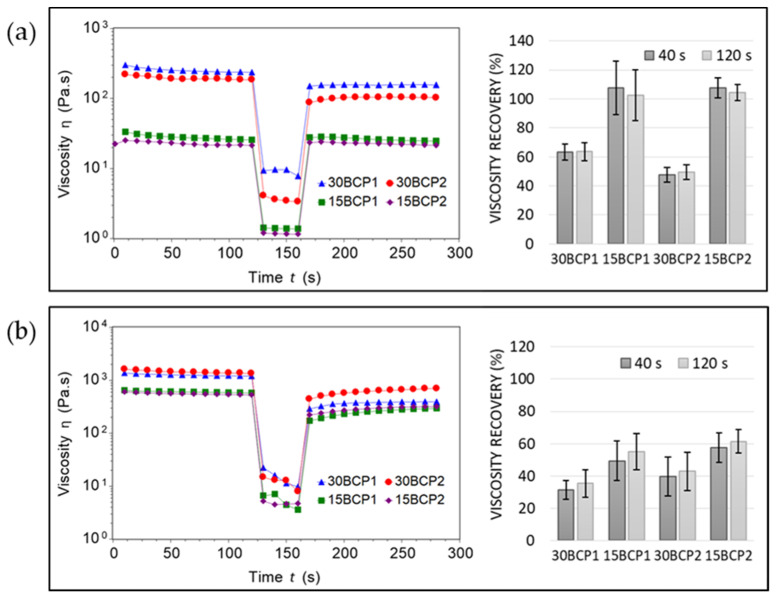
SFM test and viscosity recovery (%) at 37 °C (**a**) and 23 °C (**b**).

Tests performed at 37 °C revealed greater recovery values for inks with low particle concentration (Figure 3). However, a clear phase separation of the BCP particles from the hydrogel was observed when removing the sample from the rheometer plates. Due to the higher water content of these inks, the hydrogel was in a more liquid state at this temperature, with a diminished viscosity and internal fluid structure that hindered sustaining the BCP granules embedded in the matrix. Thus, after a high shear, the solid particles deposited in the lower layers of the sample—on the surface of the lower rheometer plate—while the hydrogel occupied the upper layers. The aggregation and compaction effect of BCP particles after a high shear resulted in viscosity recovery values higher than 100% for these inks. This behavior was also significant regarding the printing process: setting temperatures equal to or higher than 37 °C in the printhead led to a phase separation when applying a stress with the extrusion on the ink. By doing so, the hydrogel flowed freely, but the particles were retained in the nozzle, causing it to eventually clog. In this sense, SFM tests were performed at room temperature to measure the behavior of the inks in a less flowable form. In this case, the opposite phenomenon occurred: with the application of the high shear rate in the second step, samples slipped from the rheometer plates. For this reason, the recovery values obtained were low and not reliable.

**Figure 4:**
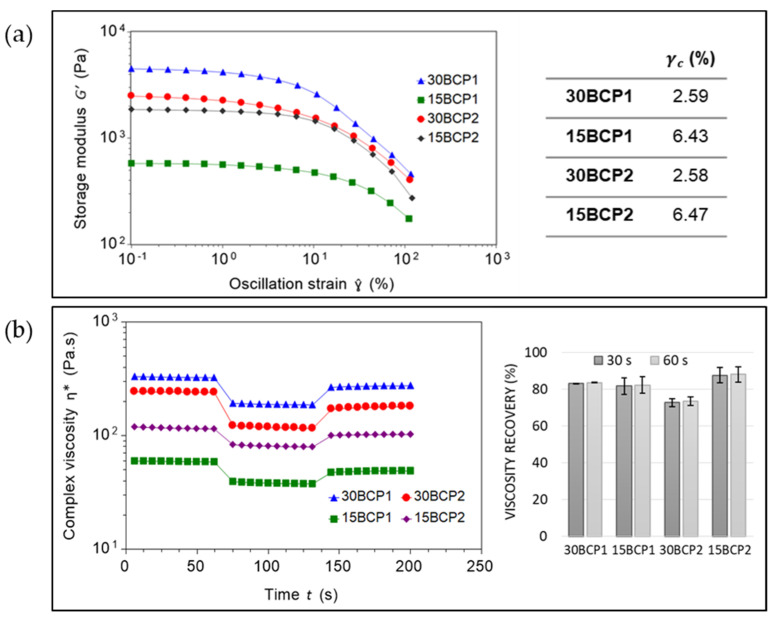
(**a**) LVR and critical strain ($\gamma_{c})$ of the inks. (**b**) Stepped Dynamic Method (SDM) and viscosity recovery (%) at room temperature.

Due to the inability to properly measure thixotropy at room temperature with the SFM test, samples were measured with an analogous assay based on low amplitude oscillation (SDM). The linear viscoelastic region (LVR) was determined to end when the shear modulus reached 90% of its initial value [45]. In general, the LVR is shortest when the sample is in its most solid form. As expected, the results obtained from the amplitude sweeps showed a smaller critical strain value $\left(\gamma_{c}\right)$ for the inks with a higher concentration of BCP (30BCP1 and 30BCP2) (Figure 4a). From this test, step two of the SDM assay was set to 20% strain to ensure the destruction of the fluid structure and correctly assess its recovery. The viscosity recovery values ranged between 72% and 89% for the different inks, which are values that allowed for a controlled deposition of the ink and a faithful execution of the models designed during 3D printing (Figure 4b). Furthermore, the samples taken from the plates after testing had a homogeneous appearance, without phase separation, thereby verifying that inks printed at temperatures around 23 °C or slightly below are maintained as a structured fluid that can withstand the application of stress during printing.

### 2.4. Evaluation of Ink Stiffness and Effect of CaCl_2_ Crosslinking

**Figure 5:**
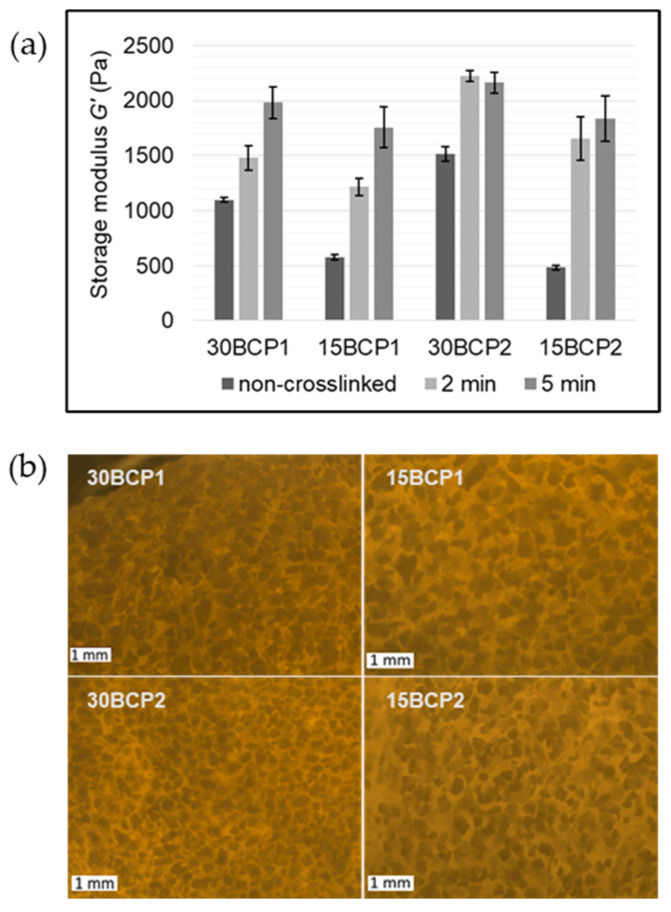
**(a)** Crosslinking effect in storage modulus (G′) values at 1% strain. (**b**) Microscope images of ink samples cast in PLA 3D-printed molds. Scale bars are equal to 1 mm.

Crosslinking the alginate present in the compositions with CaCl_2_ gives the possibility of achieving greater rigidity and malleability in the printed samples. Thus, it was necessary to evaluate the effect of the soaking time on the mechanical properties, in terms of stiffness, by oscillatory rheology [46,47,48]. To do so, samples of the different inks were casted into purposely designed 3D-printed molds and left to set at 4 °C to generate solid disks of material. Then, the disks were subjected to a range of deformations at a fixed frequency to determinate the elastic modulus (*G′*), and therefore, its stiffness (Supplementary Data, Figure S4). For all the samples, *G′* values increased with time of exposure to the CaCl_2_ solution (Figure 5a). Inks loaded with 15 wt % BCP were initially much softer, matching with the larger water content. For the same granule size, a higher particle concentration led to greater rigidity of the ink. However, *G′* rises with time in a different ratio based on the particle size. The initial increase in stiffness was more noticeable in samples with 150–250 μm BCP granules (BCP2), while for the 150–500 μm granules (BCP1), the escalation was more gradual with the exposure time.

Microscope images of the hydrogels (Figure 5b) showed that for 30 wt % BCP inks, the smaller BCP2 particles were more packed and arranged, forming a three-dimensional network with less crosslinking time. By contrast, large particles were less compact and left larger and more irregular hydrogel gaps between them, requiring more time in contact with the calcium ions to crosslink the alginate and, in turn, taking longer to generate an internal matrix that increases the stiffness of the measured sample. Inks with 15 wt % BCP have similar appearances irrespective of the particle size, with the particles more dispersed throughout the hydrogel and reaching similar stiffness values after soaking for 5 min in CaCl_2_ solution.

### 2.5. Printing Settings

**Figure 6:**
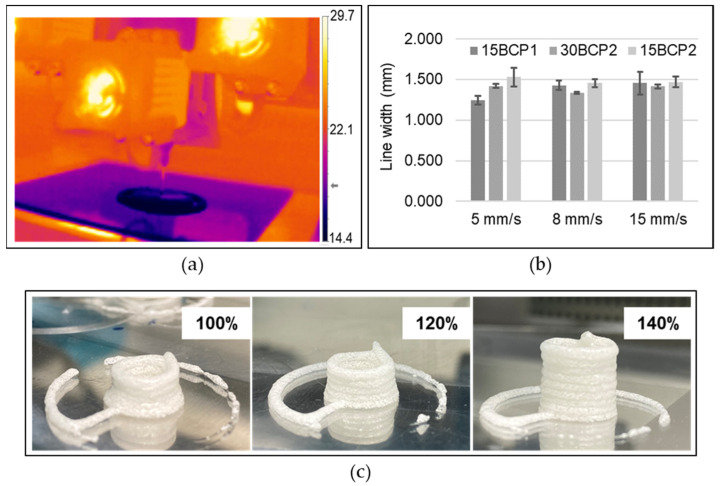
Printing set up. (**a**) Thermographic image of the temperature-induced in situ gelation of the inks. (**b**) Measured line width of filaments printed at 5, 8, and 15 mm/s. (**c**) Extrusion width calibration by printing hollow cylinders with different values of this process parameter.

The printhead and print bed temperature regulation system enabled the management of the inks’ thermo-responsive behavior by applying a controlled temperature in the syringe, which ensured the flowability without breaking the thermosensitive entanglement responsible for retaining the BCP suspension in the matrix (Figure 6a). Correct extrusion and deposition for 30 wt % ink (30BCP2) was achieved by setting the extruder temperature to 18 °C. Inks with higher water content (15BCP1 and 15BCP2) needed a slightly lower temperature in the printhead to extrude stable and regular filaments. As the flow curves predicted, printing with 30BCP1 was not suitable, as it caused nozzle clogging with the application of shear (extrusion) regardless of the temperature tested.

To select the most suitable printing speed, a simple printing test was carried out at different velocities. A speed of 8 mm/s was established for the greater definition in the lines with less deviation (Figure 6b). The extrusion width was experimentally adjusted by printing hollow cylinders. As seen in Figure 6c, the default extrusion width value led to a figure with a cylindrical design poorly reproduced. Increasing the value of this printing parameter made the printer extrude a higher amount of material. Thus, the extruded filaments formed were thicker and generated layers whose height reached the expected height layer preset by the printer. In that way, the fluid was correctly deposited on the previous layer, and a cylinder of regular diameter was generated.

### 2.6. 3D-Printed Disks: Interplay between Stiffness, Ink Composition, and Printing Process Parameters

**Figure 7:**
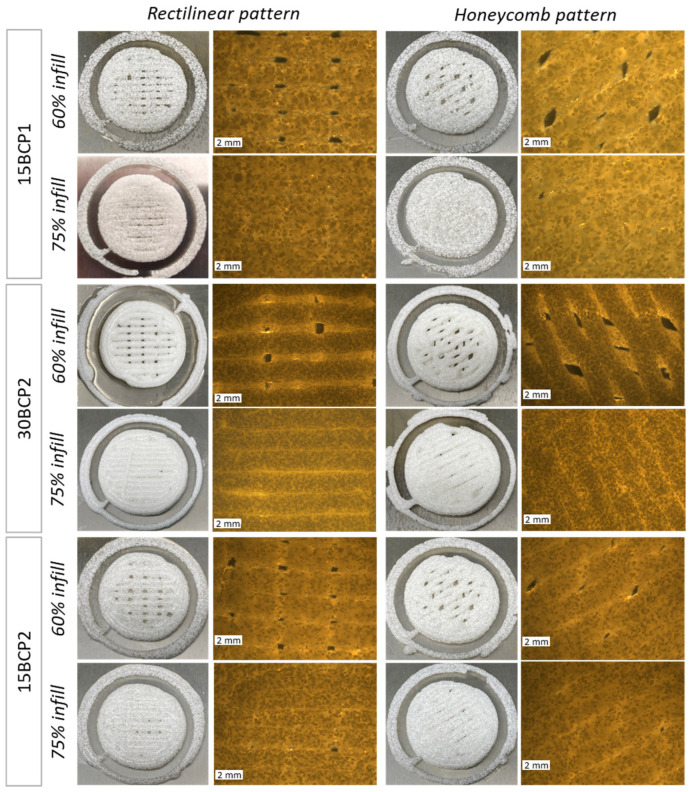
Photographic and microscopic images of disks printed with different parameters. Scale bars: 2 mm.

The four structures resulting from the combination of the printing parameters were executed correctly and precisely with the three printable inks (15BCP1, 30BCP2, and 15BCP2) (Figure 7). The skirts added in the configuration of the models fulfilled their objective of ensuring the correct flow of the ink before starting to print the desired structure, thus avoiding the appearance of defects. Microscopic observation of the figures showed that the BCP particles were uniformly distributed in the extruded filaments. Once deposited, the printed lines remained fairly defined and could be clearly distinguished, especially in the case of 30BCP2. In figures with a higher infill percentage, the deposited filaments tended to come together but did not merge completely. This result is an indicator of a good recovery of the viscoelastic properties of the inks after their extrusion, which is essential to achieve a satisfactory execution of 3D models, especially those with a substantial length in the Z-axis.

**Figure 8:**
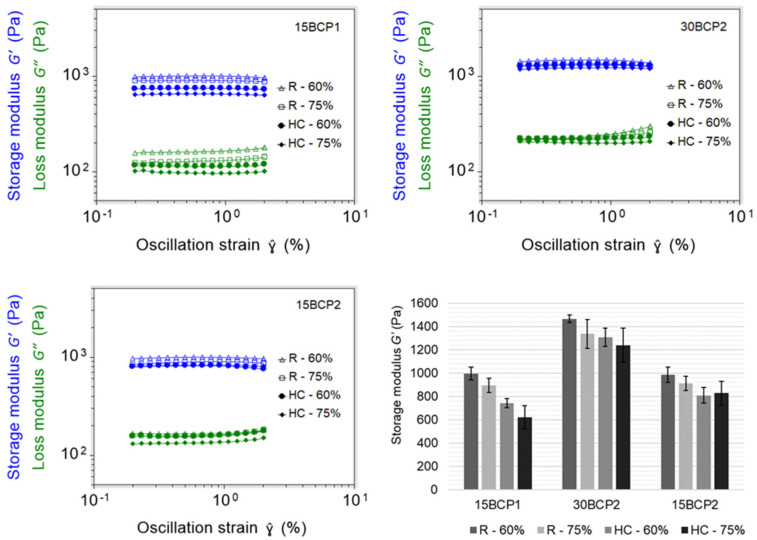
Amplitude sweep tests on 3D-printed figures and storage modulus detected at 1% strain for patterns rectilinear (R) and honeycomb (HC) at 60% or 75% infill.

Regarding the stiffness of the matrix, by comparing the values of the elastic or storage modulus (*G′*) at 1% strain, it was observed that the highest results detected corresponded in all cases to the 30BCP2 ink, regardless of the pattern or infill percentage set (Figure 8). Due to the higher concentration of solid particles, it was foreseeable that the matrices printed with this ink would have a higher value of elastic modulus, since the BCP granules enhance the biomechanical properties of the scaffold. Between different infill patterns, the figures with rectilinear filling yielded larger *G′* values. However, for the same pattern, the less filled figures had higher stiffness, since more porous structures have a greater contact surface when crosslinking with the alginate and therefore more rigidity.

Thus, it was evidenced that not only the ink composition but also the printing parameters greatly influence the biomechanical properties of the printed structures. Consequently, they must be taken into account and evaluated because, as other authors have already demonstrated, the function of cells residing in bone tissue could be affected by material stiffness [23,24,49].

### 2.7. Swelling Behavior of Printed 3D Structures

**Figure 9:**
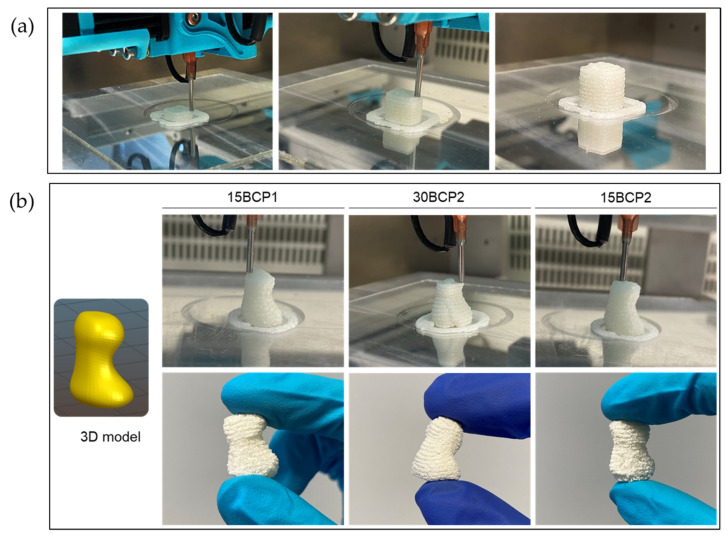
(**a**) Representative images of the printing process of cubes for swelling tests. (**b**) Phalanx model printed to evaluate the proper execution of rounded shapes. Dehydrated samples correctly retained their shape.

First, 10 mm-edge cubes were printed with 1 mm of layer height, a rectilinear pattern, and 60% infill (Figure 9a). The structures obtained successfully reproduced the 3D model both in its dimensions and appearance. A phalanx 3D model was also printed to evaluate the proper execution of rounded shapes and more complex structures (Figure 9b).

**Figure 10:**
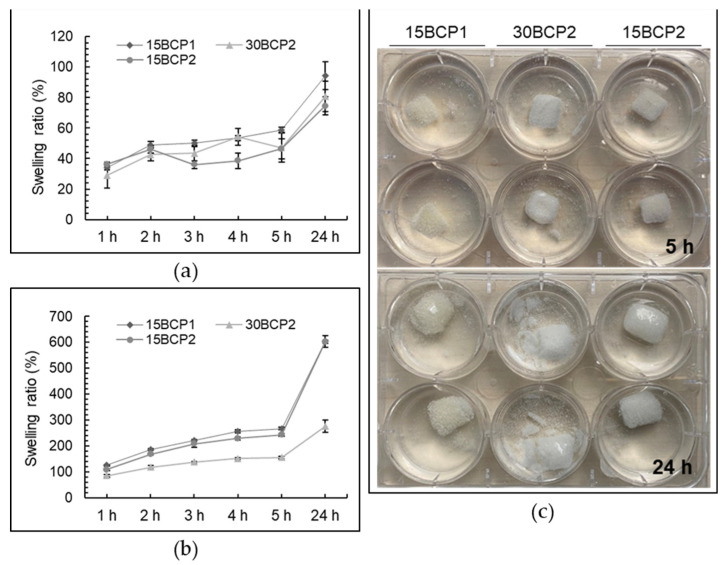
Swelling ratio (%) in DPBS as a function of incubation time of the (**a**) fresh and (**b**) dehydrated 3D-printed cubes fabricated from ceramic hydrogels of different compositions. (**c**) Optical images of the rehydrated structures after 5 h and 24 h.

The swelling ratio of the printed structures, which represents the ability of the different compositions to retain and diffuse the buffer solution, and thereby mimic the constructs ability to absorb body fluid [50], was analyzed in DPBS. In contact with the calcium-free DPBS, an exchange reaction occurred between the calcium ions of the printed structure and the sodium ions in the DPBS, breaking the crosslinks in the alginate and allowing water to enter the hydrogel [51]. For fresh samples, swelling profiles differed with respect to granule size. Figure 10a depicts that in 15BCP1 cubes, the weight (and consequently their SR%) progressively increased with the passage of minutes. However, 30BCP2 and 15BCP2 cubes showed lower SR% after 3 h of soaking due to a decrease in their weight. This result was not due to the fact that less water was captured by the hydrogel but rather because the particles contained in these inks (BCP2 granules) were released from the hydrogel matrix and deposited on the bottom of the plate already from minute 60 of soaking (Supplementary Data, Figure S7). After 24 h of incubation, the SR% for 15BCP1 cubes reached 94%, while values for 30BCP2 and 15BCP2 were only 80% and 74%, respectively, because of a higher and faster particle release with regard to the 15BCP1 samples. 

Regarding the dehydrated samples, the figures printed with 15 wt % BCP hydrogels lost almost 80% of their weight during the drying process due to its higher water content (Table 2). Consequently, figure shrinkage was also greater for 15BCP1 and 15BCP2 cubes with a reduction in the size greater than 30%. Visual appearance of dehydrated cubes can be seen in the Supplementary Data (Figure S8).

Interestingly, due to the strong hydrophilicity and swelling ability of sodium alginate and gelatin gels [52], the swelling ratio of the printed cubes increased remarkably with soaking time even after crosslinking and dehydration steps (Figure 10b). However, SR% values in time notably differed depending on the hydrogel composition: while SR% reached 600% for 15BCP1 and 15BCP2 formulations after 24 h, for 30BCP2 samples, the SR% was only 275%. As can be seen in Figure 10c, the cubes printed with 30BCP2 underwent a faster disintegration during the rehydration process. In that way, while the 15BCP1 and 15BCP2 cubes retained their shape after 24 h of soaking, the 30BCP2 samples noticeably broke down, lowering the weight of the final remaining structure and consequently reducing the SR% value. Materials with higher water absorption capacities often facilitate the wettability of the structures and subsequently enable the adhesion, proliferation, and migration of cells during the differentiation process [52]. However, this fast process of disintegration could not be suitable for cell adhesion or integration and, in this case, for lyophilized 30 wt % BCP-loaded hydrogels, stronger crosslinked matrices should be devised.

## 3. Conclusions

Due to the incidence and prevalence of osteoporosis and major trauma cases, the search for alternatives for bone repairing remains an object of interest. Among other options, 3D printing provides particular benefits such as the creation of on-demand implants tailored to the patient needs. In this vein, this work has investigated the generation of new ceramic biomaterial inks by combining a hydrocolloid-based hydrogel with BCP particles of sizes between 150 and 500 µm. After a detailed evaluation of the rheological and mechanical properties, it has been determined that both the concentration and the particle size have a significant influence on the behavior of the inks. In addition, other factors such as the crosslinking time or the configuration of the printing process parameters also imply changes in the final structure achieved, acquiring different levels of stiffness and rigidity. Likewise, apart from the matrix composition, the swelling behavior is influenced by the post-processing applied to the printed structures such as drying procedures. Thereby, the combination of all these factors generates printed structures with totally tunable stiffness, swelling, or degradation, where everything can be modulated in terms of achieving a better cell adhesion and interaction, potentiating the osteoconductivity of BCP particles, and promoting bone healing.

## 4. Materials and Methods

### 4.1. Materials

Sodium alginate (sodium salt of alginic acid from brown algae; CAS no. 9005-38-3) and gelatin (175 g Bloom, Type A, porcine skin, suitable for cell culture; CAS no. 9000–70-8) were obtained from Sigma-Aldrich (Darmstadt, Germany). Xanthan gum (CAS no.11138–66-2) was purchased from Fagron Ibérica SAU (Terrassa, Spain). Dulbecco’s Phosphate-Buffered Salt Solution 1X, without calcium and magnesium (DPBS) (Corning™ 21-031-CV) was purchased from Fisher Scientific SL (Madrid, Spain). Commercially available biphasic calcium phosphate (BCP) bone graft (MagnetOs™ Granules; Kuros Biosciences BV, Bilthoven, the Netherlands) was kindly provided as 150–500 µm size particles (BCP1) and 150–250 µm size particles (BCP2).

### 4.2. BCP-Hydrogel Preparation

Xanthan gum, sodium alginate, and gelatin mixtures were combined until obtaining a high-viscosity thermo-reversible hydrogel. Four biomaterial inks were prepared by mixing the same hydrogel with different ratios and particle sizes of BCP (Table 3). The formulations were prepared as follows. Firstly, gelatin was hydrated with deionized water and melted in a bath at 40 ± 2 °C. Meanwhile, the required amount of xanthan gum and sodium alginate powders were manually mixed with water gradually added. Once a homogeneous paste was formed, molten gelatin was slowly incorporated and gently mixed. Finally, the corresponding size and quantity of biphasic calcium phosphate granules—wetted with deionized water for an easy manipulation—were aggregated to the mix. Manual mixing was performed during the entire process and is highly recommended to avoid air incorporation into the ink. The formula was left to rest in a bath at 60 ± 2 °C for 15 min. During this time, the container was wrapped with food-grade plastic protective film to prevent water loss. Printer-compatible syringes (BD 3 mL Syringe Luer-Lok™Tip; Benton, Dickinson and Company, Aalst, Belgium) were filled, while inks remained hot and stored in the fridge at 4 °C until use. Pictures of the formulation steps are available in the Supplementary Data (Figure S1).

### 4.3. Rheological Analysis and Printability Assessment

The rheological characterization of ink samples was carried out with a controlled stress rheometer (Discovery HR-2, DHR, TA Instruments, New Castle, WI, USA) equipped with a parallel plate (25 mm diameter) and a controlled convection/radiant heating oven for stable temperature control (Environmental Test Chamber, ETC, TA Instruments, New Castle, USA). The formulations were warmed up in a 30 °C water bath for 30 min before testing to form a more flowable state that would allow the ink samples to be handled without excessively damaging their internal structure and prevent air entrapment. Samples (≈1.5 mL) were loaded with a spatula. In every test, the mean average data of three replicates were used to plot the curves. Results were recorded and processed by Trios software (Trios Rheology Software, TA Instruments, New Castle, USA).

#### 4.3.1. Flow Behavior

The shear-viscosity tests were conducted in flow ramp mode with the shear rate increasing from 0.01 to 100 s^−1^ within 120 s at 37 °C. Shear rheology was characterized by fitting the experimental data to the model of Herschel–Bulkley ($\tau =\tau_{0}+K{\left(\dot{\gamma }\right)}^{n}$), where $\tau_{0}$ is the yield stress (Pa) below which there is no flow, K is the consistency index (Pa·$s^{n}$), and n is the flow index that defines the degree of non-Newtonian behavior (shear thickening for n > 1 and shear thinning for n < 1) [42,53]. The three rheometric parameters were evaluated.

#### 4.3.2. Thixotropy and Viscosity Recovery

Thixotropy was firstly measured at 37 °C and room temperature through a shear recovery test known as the Stepped Flow Method (SFM), which consisted of 3 different steps: (1) a low shear rate of 0.4 s^−1^ for 120 s, (2) a high shear rate at 100 s^−1^ for 40 s, and finally, (3) a low shear rate of 0.4 s^−1^ for 120 s. The regeneration of the fluid’s internal structure was determined as the percentage of viscosity obtained during the first 40 s and the last 120 s in the third step (after high shear rate), based on the mean average viscosity obtained in the last 40 s of the first step, where equilibrium viscosity was reached. As SFM measurements performed at room temperature resulted in poorly reproducible results, the Stepped Dynamic Method (SDM) was instead used to properly evaluate the structural regeneration at this temperature. Firstly, the linear viscoelastic interval (LVR)—and its linearity limit ($\gamma_{c}$)—was determined by means of amplitude sweeps in a strain interval of 0.1 to 100% and at a fixed frequency of 1 Hz. SDM tests were performed to measure complex viscosity ($\eta^{*})$ under low deformation (0.1% strain), high deformation (20% strain, out of the LVR of the inks to destroy the internal structure of the samples), and again under low deformation. Complex viscosity recovery was determined as the percentage of viscosity obtained during the first 30 s and the last 60 s in the third step (after high deformation) based on the mean average viscosity obtained in the last 30 s of the first step.

### 4.4. Effect of CaCl_2_ Crosslinking on BCP-Hydrogel Matrix Stiffness

Samples of the four formulated ceramic inks were cast in custom-designed and 3D-printed molds. First, 25 × 25 × 5 mm disk-shaped molds were manufactured in-house in PLA by Fused Deposition Modeling (FDM). Pictures of the printing process and the molds can be seen in the Supplementary Data (Figure S2). Once filled with the inks, the molds were covered with film to prevent water loss and stored at 4 °C for 1 h. The crosslinking effect of CaCl_2_ on casted samples was investigated at 23 °C by performing amplitude sweeps from 0.2% to 2% strain using the same rheometric equipment described in Section 4.3. Samples were measured before crosslinking and after 2 or 5 min covered in 0.5% (*w*/*v*) CaCl_2_ solution.

### 4.5. 3D-Printing Settings

A stepper motor-driven syringe-based extrusion 3D printer (bIDO-I, Idonial Technological Center, Gijón, Spain) was used to print the constructs. For every assay, a selected 3D model was imported into an open-source slicing software (Slic3r), from which different versions of G-code were exported depending on the printing parameters set. Stainless steel, blunt-end dispenser tips (Fisnar, Glasgow, United Kingdom) with a 1.37 mm inner diameter (15G) were used as printer nozzles. To achieve a correct flow, the extruder temperature was set to 18 °C for 30BCP2 and 16 °C for 15BCP1 and 15BCP2 inks. The printing bed temperature was adjusted to 15 °C to ensure the complete gelation of the deposited material. A thermographic camera (Optris^®^ PI 230; Optris GmbH, Berlin, Germany) allowed for the visualization of the fluid-to-solid ink transition phase. Flat glass pieces were used as a support to remove the figures easily from the printing bed, facilitate cleaning tasks, and reduce the waiting time between printing processes.

#### 4.5.1. Printing Speed Selection

To select a suitable printing speed, simple squares were printed in triplicate at three different speeds (5 mm/s, 8 mm/s, and 15 mm/s). The uniformity and thickness of the printed struts were measured underneath the microscope to assess the most appropriate velocity.

#### 4.5.2. Extrusion Width Calibration

The extrusion width is the thickness of a single filament extruded either in free air or above a surface. Adjusting this printing parameter can improve the performance of the printing process and thus obtain far more satisfactory results. For that purpose, a hollow cylinder was printed while increasing the extrusion width value until the figure was properly executed.

### 4.6. Stiffness of Figures with Different Structural Configurations

The relative stiffness of 3D-printed disks (25 × 25 × 2 mm) resulting from the combination of each BCP-hydrogel with two main printing parameters (infill density and infill pattern) was assessed.

#### 4.6.1. 3D Model Setup

**Figure 11:**
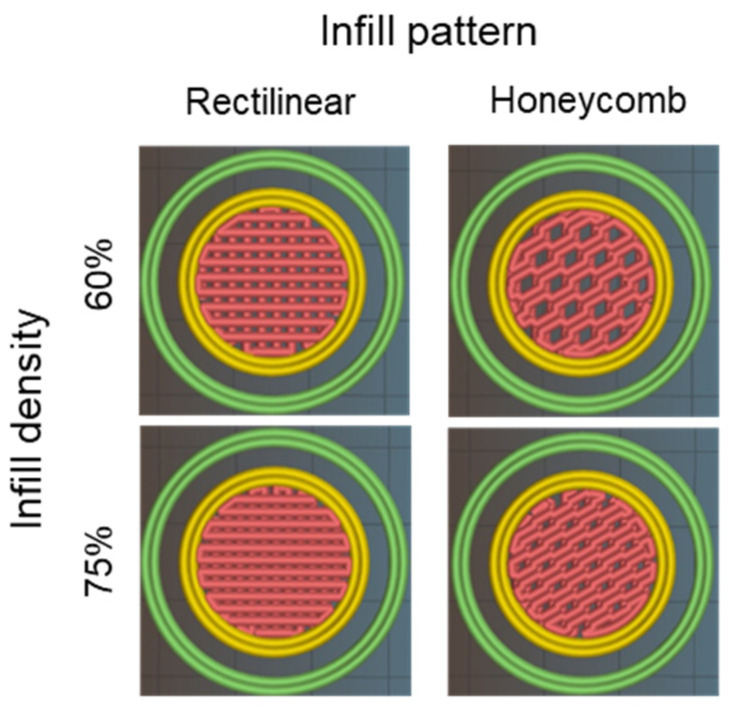
Slic3r software preview of disk-shaped 3D model with different printing process parameters configured.

By combining two infill patterns (rectilinear and honeycomb) with two infill densities (60% and 75%), four different structures (Figure 11) were configured to be tested with printable inks (15BCP1, 30BCP2, and 15BCP2). A double skirt was added to establish continuous ink flow before figure printing.

#### 4.6.2. Stiffness Measurement of the Printed Figures

The stiffness of the printed matrices was measured by performing amplitude sweeps from 0.2% to 2% strain, similarly to Section 4.4. Samples were soaked in a 0.5% (*w/v*) CaCl_2_ solution for 2 min and then washed twice with deionized water before the test.

### 4.7. Swelling Degree of Fresh and Dehydrated 3D-Printed Cubes

Six cubes (10 × 10 × 10 mm) were printed with each of the ceramic-ink formulations (15BCP1, 30BCP2 and 15BCP2); then, they were crosslinked for 2 min and washed twice with deionized water. Samples were divided in two groups for subsequent tests. The swelling ratio (SR%) of BCP hydrogels was calculated as follows:

$$SR\%=\frac{\left(W_{f}-W_{i}\right)}{W_{i}}\;x\;100$$

where $W_{i}$ and $W_{f}$ represent the sample weights before and after water absorption, respectively. Freshly crosslinked cubes (*n* = 9) were immediately immersed in a large volume of DPBS at pH 7.4 and room temperature. Then, the figures were weighed at predetermined time intervals: 1, 2, 3, 4, 5, and 24 h. The remaining cubes (*n* = 9) were allowed to dry at room temperature and weighed until a stable weight was determined. Figure dimensions were also surveyed to evaluate structure shrinkage resulting from the dehydration process. Then, the rehydration process and SR% determination were performed by soaking the dehydrated cubes in DPBS and weighing them at the same predetermined time intervals mentioned before.

### 4.8. Microscopy

BCP raw granules and 3D-printed structures were imaged using a stereo light microscope Leica M205A equipped with a DFC295 camera (Leica Microsystems Ltd., Wetzlar, Germany).

## Figures and Tables

**Table 1:** The Herschel–Bulkley parameters for the different inks.

	$\tau_{0}\;(\mathrm{Pa})$	K (Pa.s)	n	R²
30BCP1	142.08	93.25	0.54	0.9999
15BCP1	21.92	4.98	0.73	1.0000
30BCP2	125.46	70.38	0.62	0.9993
15BCP2	15.38	3.66	0.77	0.9999

**Table 2:** Weight (W) and height (H) loss of dehydrated 3D-printed cubes.

	W_i_ (g) ± SD	W_f_ (g) ± SD	Weight lost (%) ± SD	H_i_ (mm) ± SD	H_f_ (mm) ± SD	Height lost (%) ± SD
15BCP1	1.380 ± 0.01	0.286 ± 0.01	79.09 ± 0.66	101.00 ± 1.00	69.67 ± 0.58	31.03 ± 0.34
30BCP2	1.484 ± 0.06	0.522 ± 0.02	64.36 ± 0.76	100.33 ± 0.58	93.67 ± 1.15	6.00 ± 1.49
15BCP2	1.350 ± 0.01	0.285 ± 0.01	78.87 ± 0.40	101.00 ± 1.00	68.0 ± 0.00	32.67 ± 0.67

Subscript letters “i” and “f” mean “initial” and “final”, respectively.

**Table 3:** Biomaterial ink composition.

	wt %			
	30BCP1	15BCP1	30BCP2	15BCP2
Xanthan gum	3	3	3	3
Sodium alginate	2	2	2	2
Gelatin	2	2	2	2
BCP1 (150–500 µm)	30	15	-	-
BCP2 (150–250 µm)	-	-	30	15
Deionized water	63	78	63	78

## Data Availability

The datasets generated during and/or analyzed during the current study are available from the corresponding author on reasonable request.

## Supplementary Materials

The following are available online at https://www.mdpi.com/article/10.3390/gels8010028/s1. Figure S1: (A) XG and SA mixture with deionized water. (B) Incorporation of GA to the previous mixture. (C) Wetted BCP particles. (D) Final ink with BCP included. (E). Stable and homogenous biomaterial–ink loaded in printer-compatible syringe. Figure S2: (A). FDM printing of the molds. (B). Final mold detail. (C). Sample casted and protected with film, ready to be stored at 4 ºC. Figure S3: Flow curves. Figure S4: Amplitude sweeps for stiffness determination of samples non-crosslinked (A) and after 2 min (B) or 5 min (C). Figure S5: Printed disk simple placed between rheometer plates for stiffness test. Figure S6: Fresh printed cubes. Figure S7: Swelling behavior of fresh printed cubes. Higher particle release for 150–250 µm inks (30BCP2 and 15BCP2) can be seen from min 60. Figure S8: Visual appearance of dehydrated cubes. Shrinkage is notably higher in 15BCP1 and 15BCP2 samples due to its larger water content in both formulas with regard to 30BCP2. Table S1: Line width at different printing speeds tested. Table S2: Weight and height of dehydrated 3D-printed cubes. Video S1: Extrusion width calibration, Video S2: Disk printing process.
