# Supplementary material for: Effect on Rheological Properties and 3D Printability of Biphasic Calcium Phosphate Microporous Particles in Hydrocolloid-Based Hydrogels
Source: Gels. 2022 Jan 2;8(1):28. doi: 10.3390/gels8010028 (PMC8774978; doi:10.3390/gels8010028)
Supplement: Supplementary file 1 [file gels-08-00028-s001.zip › gels-1515700-supplementary.pdf]

gels

Supplementary

Effect on Rheological Properties and 3D Printability of Biphasic Calcium Phosphate Microporous Particles in Hydrocolloid-Based Hydrogels

Helena Herrada-Manchón [1,2,*], David Rodríguez-González [1,2], Manuel Alejandro Fernández [2], Nathan William Kucko [3], Florence Barrère-de Groot [3] and Enrique Aguilar [1]

1 Centro de Innovación en Química Avanzada (ORFEO-CINQA), Departamento de Química Orgánica e Inorgánica, Instituto Universitario de Química Organometálica "Enrique Moles", Universidad de Oviedo, C/Julián Clavería 8, 33006 Oviedo, Spain; davidrgezglez@gmail.com (D.R.-G.); eah@uniovi.es (E.A.)
2 Fundación Idonial, Parque Científico y Tecnológico de Gijón, Avda, Jardín Botánico 1345, 33203 Gijón, Spain; alejandro.fernandez@idonial.com
3 Kuros Biosciences BV, Professor Bronkhorstlaan 10, Building 48, 3723 MB Bilthoven, The Netherlands; Nathan.Kucko@kurosbio.com (N.W.K.); florence.de.groot@kurosbio.com (F.B.-d.G.)
* Correspondence: helena.herrada@idonial.com

Citation: Herrada-Manchón, H.; Rodríguez-González, D.; Fernández, M.A.; Kucko, N.W.; Barrère-de Groot, F.; Aguilar, E. Effect on Rheological Properties and 3D Printability of Biphasic Calcium Phosphate Microporous Particles in Hydrocolloid-Based Hydrogels. *Gels* **2022**, *8*, 28. https://doi.org/10.3390/gels8010028

Academic Editor: Yongsung Hwang

Received: 2 December 2021
Accepted: 27 December 2021
Published: 2 January 2022

Publisher's Note: MDPI stays neutral with regard to jurisdictional claims in published maps and institutional affiliations.

Copyright: © 2022 by the authors. Licensee MDPI, Basel, Switzerland. This article is an open access article distributed under the terms and conditions of the Creative Commons Attribution (CC BY) license (https://creativecommons.org/licenses/by/4.0/).

1. Formulation Steps

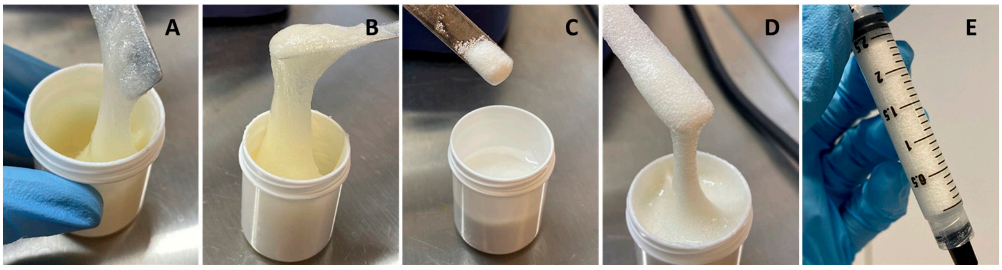

Figure S1. (**A**) XG and SA mixture with deionized water. (**B**) Incorporation of GA to the previous mixture. (**C**) Wetted BCP particles. (**D**) Final ink with BCP included. (**E**). Stable and homogenous biomaterial-ink loaded in printer-compatible syringe.

2. FDM 3D Printed Molds for $CaCl_2$ Effect Evaluation

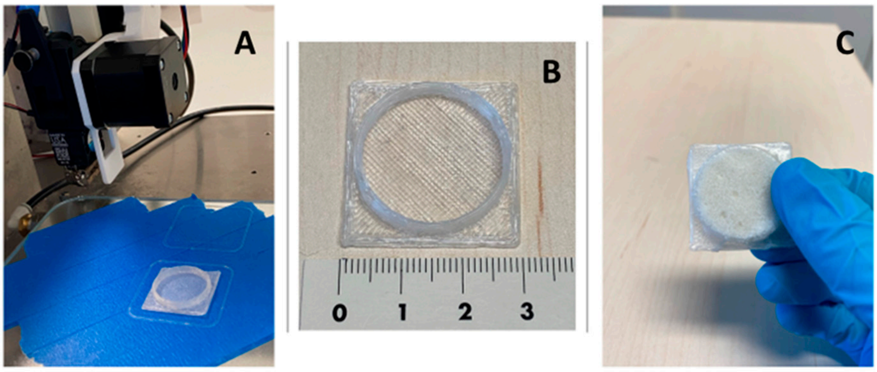

Figure S2. (**A**). FDM printing of the molds. (**B**). Final mold detail. (**C**). Sample casted and protected with film, ready to be stored at 4 °C.

3. Individual Flow Curves

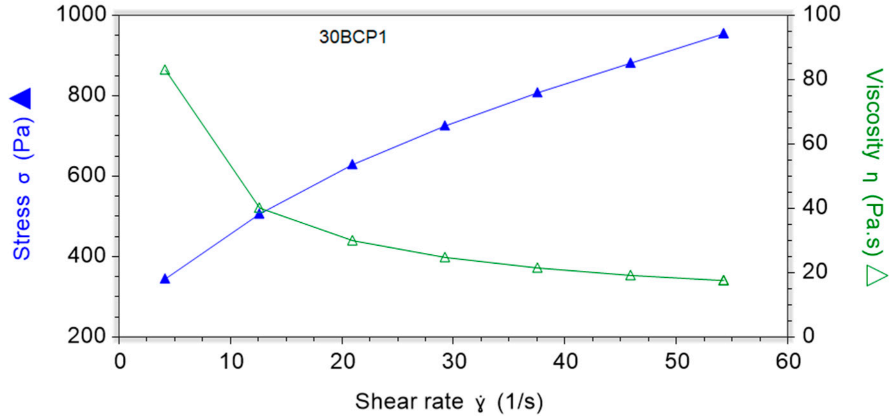

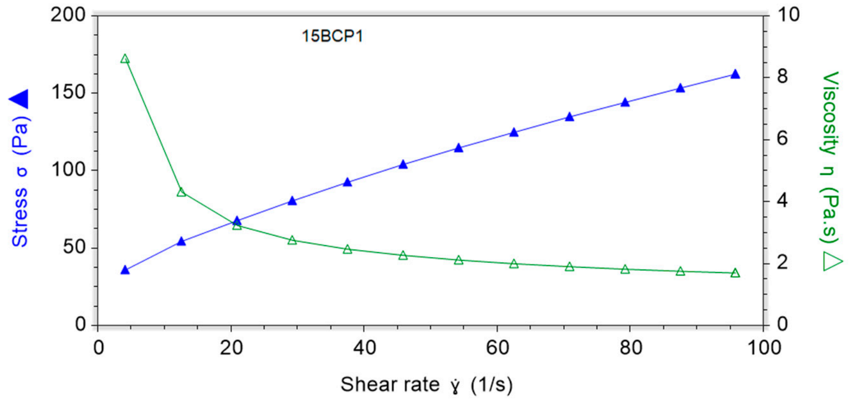

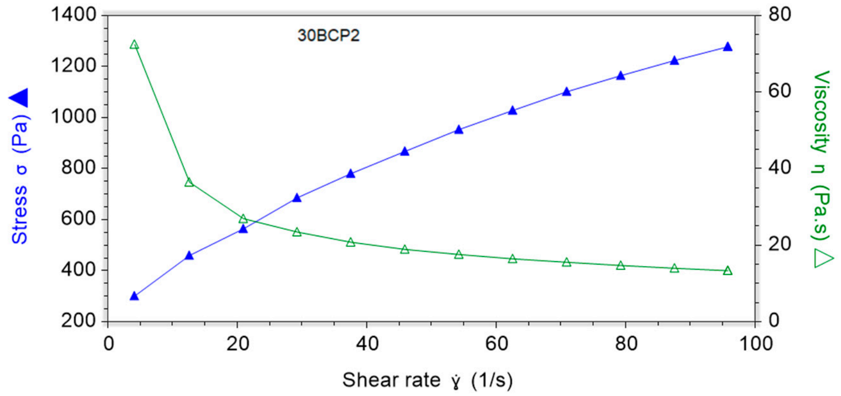

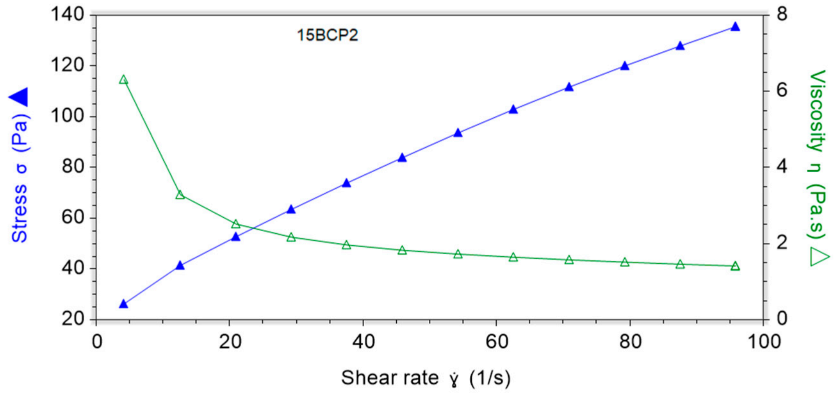

Figure S3. Flow curves.

4. Amplitude Sweeps of Casted Samples for $CaCl_2$ Effect Evaluation

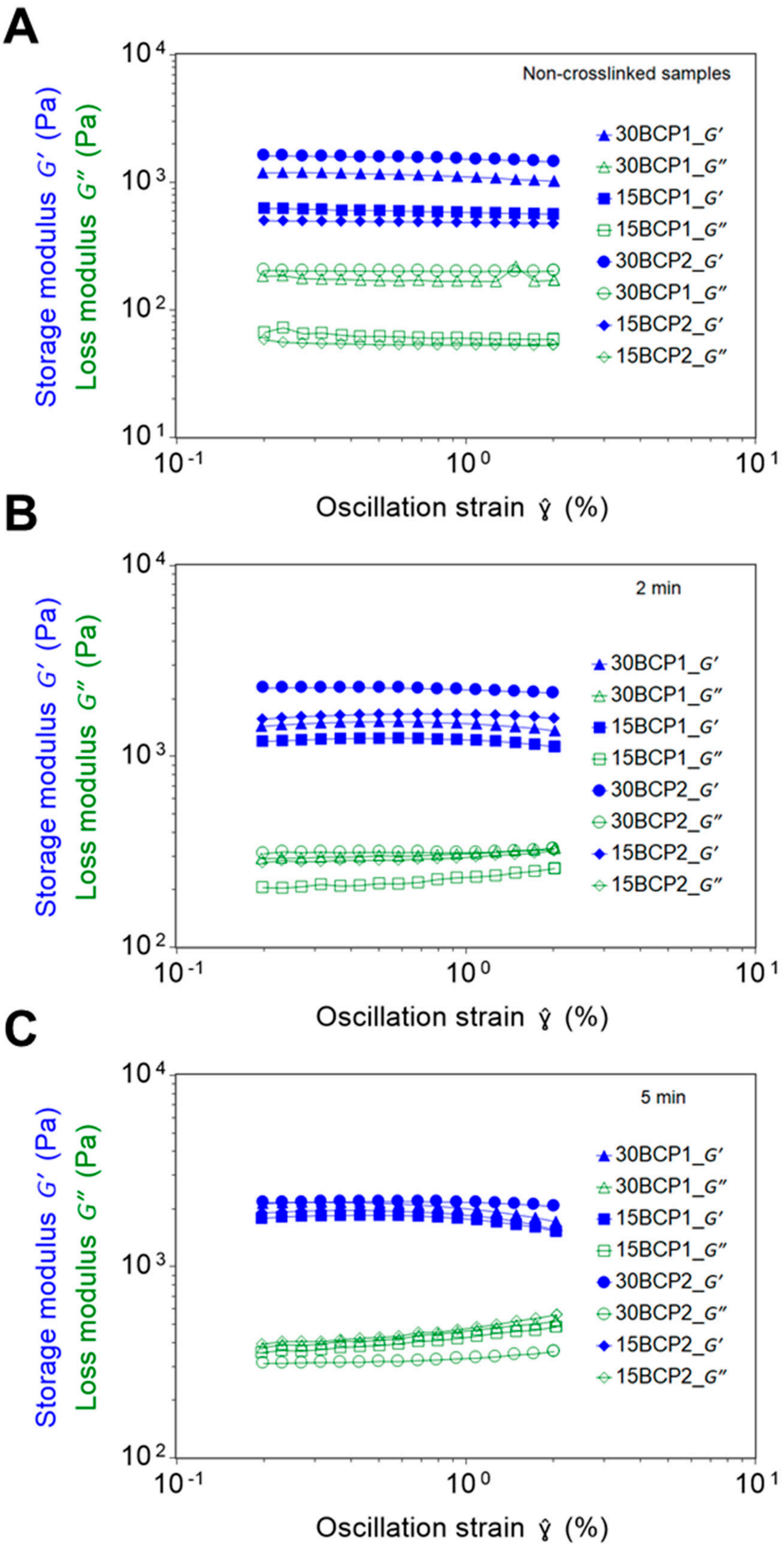

Figure S4. Amplitude sweeps for stiffness determination of samples non-crosslinked (**A**) and after 2 min (**B**) or 5 min (**C**).

5. Stiffness Measurements of Printed Figures

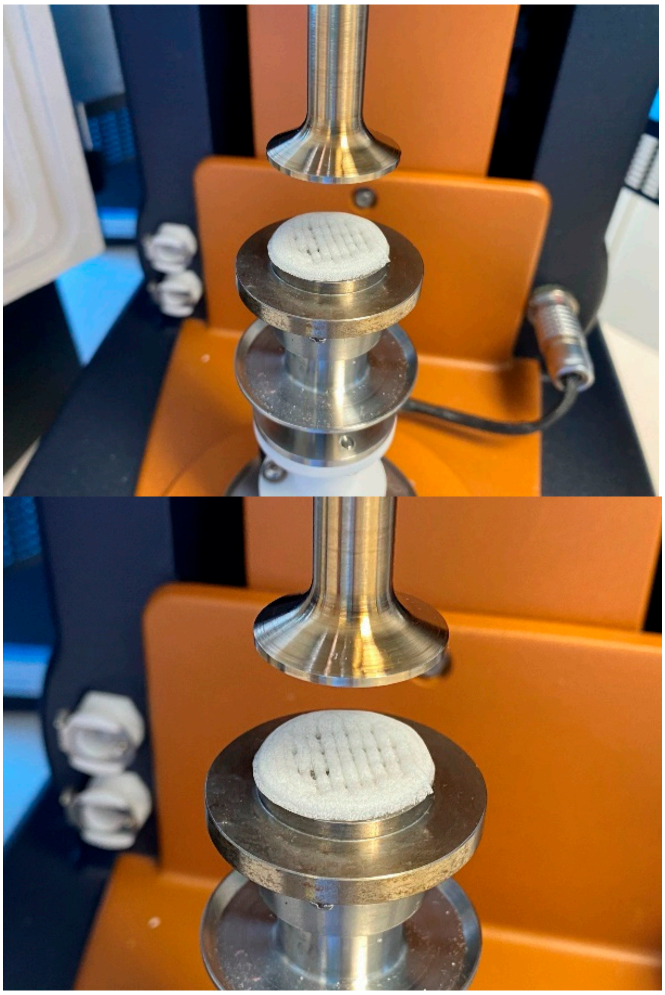

Figure S5. Printed disk simple placed between rheometer plates for stiffness test.

6. Printing Settings

Table S1. Line width at different printing speeds tested.

	5 mm/s	8 mm/s	15 mm/s
15BCP1	1.214	1.485	1.369
	1.214	1.433	1.382
	1.304	1.368	1.614
Mean	1.244	1.429	1.455
SD	0.05	0.06	0.14
30BCP2	1.445	1.350	1.383
	1.407	1.336	1.435
	1.407	1.321	1.421
Mean	1.420	1.336	1.413
SD	0.02	0.01	0.03
15BCP2	1.627	1.510	1.524
	1.549	1.446	1.394

	1.407	1.407	1.485
**Mean**	1.528	1.454	1.468
**SD**	0.11	0.05	0.07

7. 3D Printed Cubes

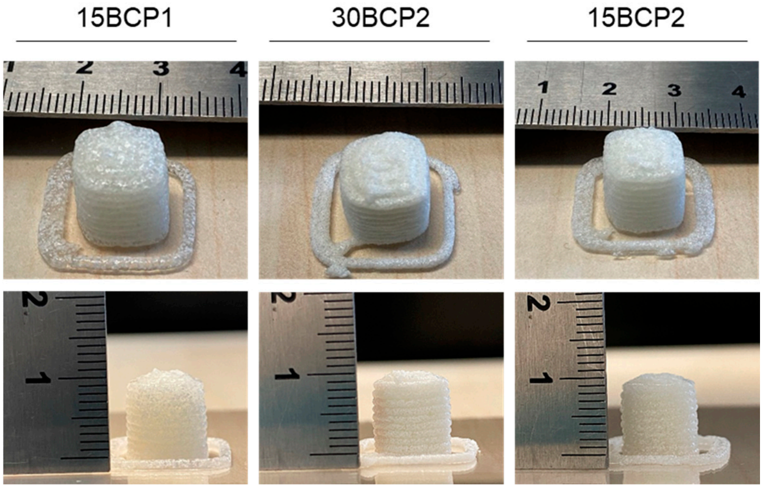

Figure S6. Fresh printed cubes.

Table S2. Weight and height of dehydrated 3D printed cubes.

	W_i (g)	W_f (g)	Weight Lost (%)	Mean ± SD	H_i (mm)	H_f (mm)	Height Lost (%)	Mean ± SD
**15BCP1**	1.391	0.283	79.65	79.09 ± 0.66	102	70	31.37	31.03 ± 0.34
	1.365	0.293	78.53		101	70	30.69	
	1.385	0.281	79.71		100	69	31.00	
**30BCP2**	1.528	0.545	64.33	64.36 ± 0.76	100	95	5.00	6.00 ± 1.49
	1.410	0.502	64.40		100	93	7.00	
	1.515	0.520	65.68		101	93	7.92	
**15BCP2**	1.345	0.278	79.33	78.87 ± 0.40	101	68	32.67	32.67 ± 0.67
	1.351	0.289	78.61		100	68	32.00	
	1.355	0.289	78.67		102	68	33.33	

W means figure weight; H means figure height.

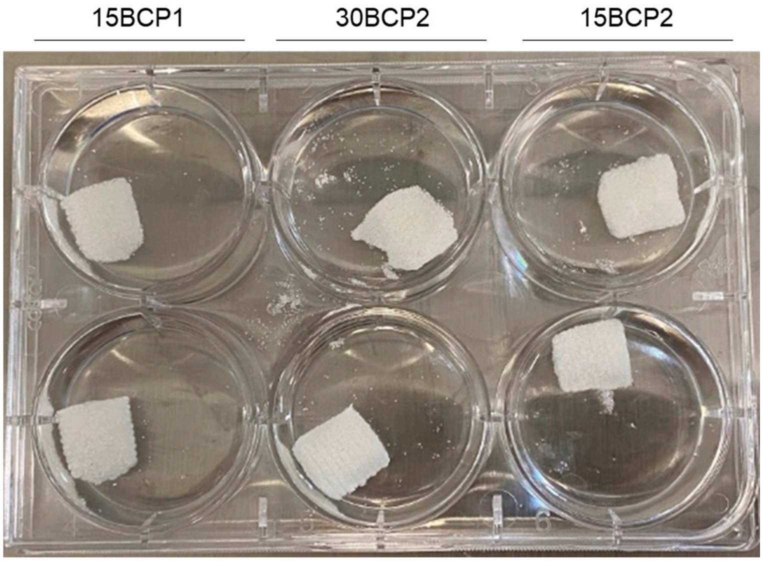

Figure S7. Swelling behavior of fresh printed cubes. Higher particle release for 150–250 μm inks (30BCP2 and 15BCP2) can be seen from 60 min.

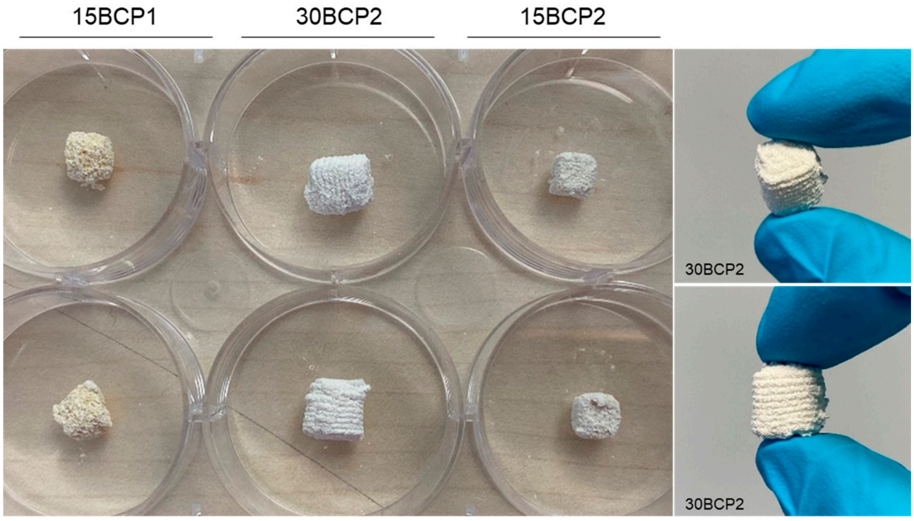

Figure S8. Visual appearance of dehydrated cubes. Shrinkage is notably higher in 15BCP1 and 15BCP2 samples due to its larger water content in both formulas with regards to 30BCP2.
